# Association Between Albumin Corrected Anion Gap and 28‐Day All‐Cause Mortality in Patients With Acute Respiratory Failure in ICU: A Retrospective Study Based on the MIMIC‐IV Database

**DOI:** 10.1111/crj.70100

**Published:** 2025-07-09

**Authors:** Jianmin Qu, Xiahong Tang, Yi Cheng, Wei Xiong, Yunfeng Zhao

**Affiliations:** ^1^ Department of Intensive Care Unit Tongxiang First People's Hospital Tongxiang Zhejiang Province China; ^2^ Department of Pulmonary and Critical Care Medicine, Xinhua Hospital Shanghai Jiaotong University School of Medicine Shanghai China; ^3^ Department of Cardiovascular Medicine, Graduate School of Medicine Kyoto University Kyoto Japan; ^4^ Department of Pulmonary and Critical Care Medicine Punan Hospital, Pudong New Area Shanghai China

**Keywords:** ACAG, ARF, linear, MIMIC‐IV, mortality, positive correlation

## Abstract

**Background:**

For critically ill patients in the intensive care unit (ICU), acute respiratory failure (ARF) stands as a prominent cause of mortality. Anion gap (AG) denotes the disparity between unmeasured cations and anions. Adjusting AG for albumin levels results in the albumin corrected anion gap (ACAG), which provides a more accurate representation of the body's acid–base status. Elevated ACAG may arise from ARF‐induced cellular hypoxia and metabolic acidosis. However, limited research has investigated the association between ACAG and the 28‐day all‐cause mortality of ARF patients in critical care.

**Methods:**

Using the Medical Information Mart for Intensive Care (MIMIC‐IV 2.2) database, a retrospective data analysis was conducted, specifically targeting critically ill patients diagnosed with ARF. Serum ACAG was collected within 24 hours of the patient's admission to the ICU. The association between ACAG levels and 28‐day all‐cause mortality was investigated using smooth curve fitting, a multivariate Cox proportional hazard regression model, and Kaplan–Meier survival curve analysis. Furthermore, the consistency of these relationships was assessed through interaction and subgroup analyses.

**Results:**

The study involved the enrollment of 3888 eligible participants in total. After adjusting for confounding variables in the multivariable Cox regression analysis model, we noticed a positive linear relationship between the ACAG value and the ICU's 28‐day all‐cause mortality rate. When ACAG was used as a continuous variable, a 3.1% increase in 28‐day all‐cause mortality was associated with a 1.0‐mmol/L increase in ACAG (adjusted HR = 1.037, 95% CI: 1.025–1.048, *p* < 0.001). In the 28‐day all‐cause mortality, the highest and intermediate ACAG groups (adjusted HR 1.483, 95% CI: 1.244–1.768 and adjusted HR 1.244, 95% CI: 1.062–1.457, respectively) were notably higher than the lowest ACAG group when ACAG was utilized as a tertiles categorical variable. The substantial association between ACAG and 28‐day all‐cause mortality in the ICU was consistently demonstrated through subgroup analysis.

**Conclusions:**

Among ICU patients with ARF, an elevated ACAG is linked to an elevated risk of 28‐day all‐cause mortality. There exists a linearly positive relationship between the 28‐day all‐cause mortality and ACAG.

## Graphical abstract

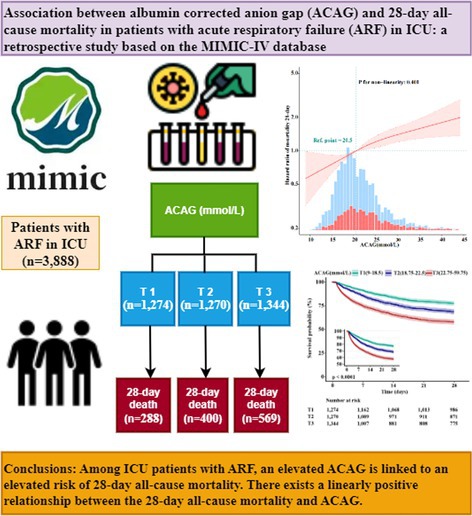

**Abbreviations**
ACAG: albumin corrected anion gap
AECOPD: acute exacerbations of chronic obstructive pulmonary disease
APSIII: acute physiology score III
BMI: body mass index
CHF: congestive heart failure
CI: confidence interval
DM: diabetes mellitus
FiO_2_
: fraction of inspiration oxygen
HA: human albumin
HB: hemoglobin
HR: hazard ratio
HTN: hypertension
ICU: intensive care unit
IMV: invasive mechanical ventilation
OASIS: oxford acute severity of illness score
OPTICS: clustering structure identification method based on ordering points
PaCO_2_
: partial pressure of carbon dioxide in arterial blood
PaO_2_
: partial pressure of oxygen in arterial blood
pH: potential of hydrogen
PF ratio: PaO_2_/FiO_2_ ratio
Plt: platelet
Ref: reference
SAPSII: simplified acute physiology score II
SOFA: sequential organ failure assessment
T: tertiles
WBC: white blood cell count

## 1 Introduction

Acute respiratory failure (ARF) is a complex and severe pathophysiological condition characterized by acute and progressive hypoxemia, often occurring in patients who were previously healthy. It can develop rapidly within hours or days, or even over the course of a month, and may be triggered by various cardiovascular, respiratory, or systemic diseases [1]. The anion gap (AG) is a widely used biomarker for diagnosing and prognosing acid ‐ base imbalances, reflecting the difference between unmeasured anions and cations in the plasma [2]. However, hypoalbuminemia, which is prevalent among critically ill patients, can mask an elevated AG [3]. Given the critical role of AG in assessing acid ‐ base status, its correction for hypoalbuminemia is essential to accurately reflect the underlying metabolic state.

Albumin, a major plasma protein, has multiple physiological functions, including maintaining plasma colloid osmotic pressure, regulating acid–base balance, and exerting antioxidant and anti‐inflammatory effects [4]. Its negative charge can influence plasma anion concentration [5]. Hypoalbuminemia reduces protein anions in plasma, thereby lowering the AG and potentially masking metabolic acidosis [6]. The albumin corrected anion gap (ACAG) adjusts for albumin levels to provide a more accurate reflection of changes in unmeasured anions, thus avoiding misjudgment of acid–base status [7]. Previous studies have demonstrated that a higher ACAG is independently associated with increased hospital mortality in patients with acute pancreatitis [8] and shows superior predictive capability for mortality in sepsis patients compared to albumin and AG alone [9].

Despite these insights, the association between ACAG and 28‐day all‐cause mortality in ARF patients remains underexplored. Therefore, this study aims to investigate the relationship between serum ACAG and 28‐day all‐cause mortality in ICU patients with ARF.

## 2 Methods

### 2.1 Data Sources and Setting

The Medical Information Mart for Intensive Care‐IV database, specifically version 2.2 (MIMIC‐IV), was employed in this retrospective observational cohort analysis. Clinical data of patients admitted to Beth Israel Deaconess Medical Center (BIDMC) from 2008 to 2019 was obtained through MIMIC‐IV, an openly accessible real‐world database [10]. From 2008 to 2019, the MIMIC‐IV 2.2 database recorded a total of 73, 181 ICU admissions. Qu J granted permission for the utilization of the database (certification number 42668161). The Institutional Review Boards (IRBs) of the Massachusetts Institute of Technology (MIT) (No. 0403000206) and BIDMC (No. 2001‐P‐001699/14) granted authorization for the data's usage in research after its de‐identification. The use of information in our study has been carried out in strict accordance with all relevant ethical standards.

### 2.2 Study Population

In accordance with the International Classification of Diseases, Ninth Revision (ICD‐9) and Tenth Revision (ICD‐10), our study incorporated individuals diagnosed with ARF. Acknowledging that ARF may not consistently signify the primary diagnosis, we also incorporated records where ARF appeared within the first five diagnostic positions of the sequence [11]. ARF diagnosis relied on the presence of at least one of the following criteria: (1) arterial blood partial pressure of oxygen (PaO_2_) less than 60 mmHg; (2) PaO_2_/FiO_2_ ratio < 300 mmHg (arterial oxygen partial pressure/inspired oxygen fraction); (3) requirement for ventilator support. The inclusion criteria were as follows: (1) Patients diagnosed with ARF, including those with International Classification of Diseases, Ninth Revision, Clinical Modification (ICD‐9‐CM) codes (51 881, 51 851) and Tenth Revision, Clinical Modification (ICD‐10‐CM) codes (J960, J9,600, J9,601, J9,602). (2) Main diagnosis records with ARF listed in any of the first five diagnostic positions. (3) Patients with ARF received treatment in the ICU. The exclusion criteria were as follows: (1) ARF patients with ICU stays < 24 hours; (2) patients with missing key variables (AG and albumin); (3) data from patients with second or subsequent ICU admissions.

### 2.3 Exposure Variable

The data for this study were extracted exclusively from MIMIC‐IV 2.2 databases using Structured Query Language (SQL) and Navicat Premium software (version 15.0.12). The AG and serum albumin were collected 6 hours prior to and 24 hours following the ICU admission. AG was extracted of which the itemids were 50 868 and 52 500. Following the extraction of albumin, the itemids were 50 862, 53 085, and 53 138. The following formula was used to calculate the AG: AG (mmol/l) = (sodium + potassium) − (chloride + bicarbonate). Additionally, ACAG was determined with the following formula: ACAG (mmol/L) = [4.4‐ (albumin(g/dL))] * 2.5 + AG [12].

### 2.4 Covariates

We chose the pertinent factors for the current study from an earlier article [13]. These factors were frequently considered in research on the relationship between ACAG and outcome.

The following variables were used:

ICU Admission Information: Age, gender, race, weight, height, body mass index (BMI), and comorbidities including congestive heart failure (CHF), acute exacerbation of chronic obstructive pulmonary disease (AECOPD), diabetes mellitus (DM), and hypertension (HTN).
Severity of Disease: Acute physiology score III (APSIII), Simplified Acute Physiology Score II (SAPS II), Oxford Acute Severity of Illness Score (OASIS), and Sequential Organ Failure Assessment (SOFA).
Laboratory Test: Select the following data: the highest values of white blood cell count (WBC), glucose, creatinine, potassium, sodium, lactate, and partial pressure of carbon dioxide in arterial blood (PaCO₂), as well as the lowest values of hemoglobin (HB), platelet (Plt), albumin, AG, calcium, potential of hydrogen (pH), partial pressure of oxygen in arterial blood (PaO₂), and the PaO₂/FiO₂ ratio (PF ratio), within the 6 h before and the 24 h after ICU admission, if the patient has multiple laboratory tests.
Organ Support and Treatment: Data regarding the use of invasive mechanical ventilation(IMV), vasoactive agents, and human albumin (HA) within the 6 h preceding ICU admission and the 24 h following ICU admission.
Outcome: The primary endpoint of our study was 28‐day all‐cause mortality. Patients who were discharged after 28days were considered alive in the hospital. The survival time was obtained by subtracting the time of admission to the ICU from the time of death. The patient's date of death was subtracted from the ICU admission time if there was no death time. “Death” was defined as a survival time of less than or equal to 28days, and “survival” was defined as a survival time of more than 28days.

### 2.5 Statistical Analysis

Based on the ACAG tertiles, patient characteristics and demographics were represented. Counts and percentages were used to display categorical variables. For continuous variables with a normal distribution, the means and standard deviations (SD) were presented; for skewed distributions, the median and interquartile range (IQR) were presented. We utilized one‐way ANOVA for variables exhibiting a normal distribution, the Kruskal–Wallis *H* test for those with a skewed distribution, and chi‐square tests for categorical variables. These methods were employed to evaluate statistical differences in means and proportions among the groups.

We conducted both univariate Cox regression analyses and multivariable Cox regression analyses to assess the association between covariates, including ACAG and 28‐day all‐cause mortality. Confounder screening was performed based on the following criteria: (1) Variables with *p*‐values < 0.20 from the univariate analysis were incorporated into the initial model. (2) Confounder selection employed a stepwise backward method, retaining only variables with *p*‐values < 0.05 in the multivariable models. (3) Other variables with clinical significance or had a substantial impact (more than 10%) on the research variable were also kept in the final model.

We simultaneously showed the results of the Crude Model (without adjustment for any covariates); Model I adjusted analyses (age, race, BMI, and gender); Model II; we adjusted Model I plus CHF, AECOPD, DM, HTN, APSIII, SAPIII, OASIS, and SOFA; Model III adjusted analyses Model II plus HB, WBC, Plt, glucose, creatinine, lactate, pH, PaO_2_, PaCO_2_, PF ratio, IMV, and vasoactive agent); Model IV adjusted analyses Model III plus HA infusion on the first day of ICU admission.

Furthermore, we utilized a generalized additive model (GAM) to explore linear correlations. When ACAG and 28‐day all‐cause mortality appeared evident in the smoothed curve, by including the tertile values of each ACAG category as a continuous parameter in the models, we were able to conduct linear trend tests.

We performed multiple imputations in accordance with the Monte Carlo approach to mitigate any bias due to missing data, producing five complete datasets [14]. Statistical analyses were conducted using the R software (http://www.R‐project.org, The R Foundation) and Free Statistics software version 1.9.2 [15] to derive all results. *P* values below 0.05 (two‐sided) were considered to indicate statistical significance.

### 2.6 Sensitivity analysis

We conducted sensitivity analyses using the entire dataset, inclusive of values that had been excluded due to missing data.

## 3 Results

### 3.1 Baseline Demographics and Characteristics of Patients

**FIGURE 1:**
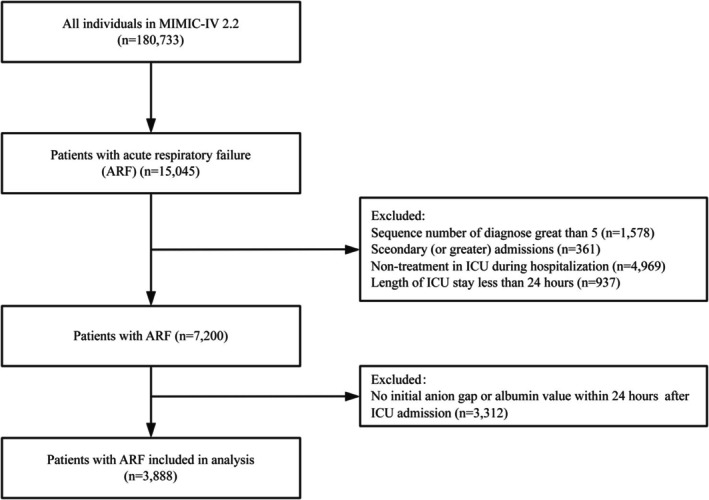
Flowchart of the study. ARF, acute respiratory failure; ICU, intensive care unit; MIMIC‐IV, Medical Information Mart for Intensive Care.

After the selection of participants from a total of 73,181 MIMIC‐IV 2.2 admissions, a total of 15,045 patients with ARF were identified and included in the study. Ultimately, 3,888 individuals with complete ARF data entered the final analysis set following the exclusion of ineligible patients (Figure 1).

The baseline characteristics are presented based on ACAG tertiles for the patients: Low group (T1): 9–18.5 mmol/L; Intermediate group (T2): 18.75–22.5 mmol/L; High group (T3): 22.75–59.75 mmol/L. The mean age of the participants was 64.5 ± 16.8 years, whereas 56.4% of the patients were male. There were statistically significant differences in gender, age, CHF, HTN, DM, SOFA score, APSIII score, OASIS score, SAPSII score, HB, WBC, Plt, albumin, creatinine, glucose, calcium, potassium, lactate, PaO_2_, PaCO_2_, PF ratio, IMV, and vasoactive agents among the different ACAG groups (Table 1).

**TABLE 1:** The clinical characteristics of critically ill patients with ARF according to ACAG levels.

Characteristics	ACAG (mmol/L)	ACAG mmol/L (tertiles)			
	Total (*n* = 3888)	T1 (9–18.5) (*n* = 1274)	T2 (18.75–22.5) (*n* = 1270)	T3 (22.75–59.75) (*n* = 1344)	*p*
Demographic characteristics					
Race, *n* (%)					0.459
White	2281 (58.7)	756 (59.3)	745 (58.7)	780 (58.0)	
Non‐White	1607 (41.3)	518 (40.7)	525 (41.3)	564 (42.0)	
Gender					0.004
Male, *n* (%)	2194 (56.4)	718 (56.4)	675 (53.1)	801 (59.6)	
Age (years)	64.5 ± 16.8	63.6 ± 17.0	65.6 ± 17.2	64.2 ± 16.1	0.008
BMI (kg/m^2^)	28.9 ± 8.2	28.8 ± 8.4	28.8 ± 7.9	29.2 ± 8.1	0.414
Comorbidities					
CHF, *n* (%)	1276 (32.8)	373 (29.3)	448 (35.3)	455 (33.9)	0.003
AECOPD, *n* (%)	531 (13.7)	185 (14.5)	178 (14)	168 (12.5)	0.291
HTN, *n* (%)	1060 (27.3)	391 (30.7)	338 (26.6)	331 (24.6)	0.002
DM, *n* (%)	1161 (29.9)	322 (25.3)	362 (28.5)	477 (35.5)	< 0.001
Severity of disease					
SOFA	3.0 (2.0, 5.0)	3.0 (2.0, 4.0)	3.0 (2.0, 5.0)	4.0 (3.0, 6.0)	< 0.001
APSIII	62.5 (45.0, 86.0)	50.0 (37.0, 67.0)	61.0 (44.0, 82.0)	81.0 (59.0, 103.0)	< 0.001
OASIS	39.4 ± 9.4	36.4 ± 8.4	38.7 ± 8.7	42.8 ± 9.7	< 0.001
SAPSII	43.8 ± 16.0	37.0 ± 13.2	42.1 ± 13.7	51.8 ± 17.1	< 0.001
Laboratory parameters					
HB, (g/dL)	10.0 ± 2.3	10.5 ± 2.2	10.0 ± 2.2	9.6 ± 2.4	< 0.001
WBC, (× 10^9^/L)	14.1 (9.9, 19.8)	12.1 (8.9, 16.2)	14.2 (10.0, 19.6)	16.4 (11.3, 23.0)	< 0.001
Plt (× 10^9^/L)	165.0 (106.0, 232.0)	175.0 (122.0, 234.0)	171.0 (111.2, 237.0)	147.0 (79.0, 225.0)	< 0.001
Albumin, (g/dL)	3.1 ± 0.7	3.3 ± 0.7	3.1 ± 0.7	2.9 ± 0.7	< 0.001
Creatinine, (mg/dL)	1.3 (0.9, 2.2)	1.0 (0.7, 1.3)	1.3 (0.9, 1.9)	2.2 (1.4, 3.8)	< 0.001
Glucose (mg/dL)	135.0 (111.0, 173.0)	127.0 (108.7, 155.7)	134.4 (111.0, 168.2)	149.5 (116.0, 199.8)	< 0.001
Calcium (mg/dL)	7.8 ± 0.9	8.1 ± 0.8	7.8 ± 0.9	7.5 ± 1.0	< 0.001
Sodium (mmol/L)	140.6 ± 6.0	140.9 ± 4.8	140.6 ± 5.8	140.4 ± 7.1	0.148
Potassium (mmol/L)	4.8 ± 1.0	4.5 ± 0.8	4.7 ± 0.9	5.1 ± 1.1	< 0.001
Lactate (mmol/L)	3.2 ± 3.5	1.9 ± 1.5	2.5 ± 3.0	5.0 ± 4.4	< 0.001
pH	7.31 (7.2, 7.3)	7.35 (7.28, 7.40)	7.33 (7.24, 7.39)	7.25 (7.14, 7.35)	< 0.001
PaO_2_ (mmHg)	75.0 (58.0, 103.0)	80.0 (61.0, 115.0)	76.0 (59.0, 103.0)	71.0 (56.0, 93.0)	< 0.001
PaCO_2_ (mmHg)	47.5 ± 15.4	50.4 ± 17.1	46.9 ± 14.4	45.4 ± 14.2	< 0.001
PF ratio (mmHg)	154.1 (95.0, 245.0)	174.0 (109.2, 268.5)	156.0 (96.0, 251.9)	137.3 (86.2, 220.2)	< 0.001
Treatment on the first day of ICU admission					
IMV, *n* (%)	2762 (71.0)	877 (68.8)	876 (69)	1009 (75.1)	< 0.001
Vasoactive agent, *n* (%)	2171 (55.8)	520 (40.8)	694 (54.6)	957 (71.2)	< 0.001
HA, *n* (%)	1574 (40.5)	514 (40.3)	531 (41.8)	529 (39.4)	0.440
Outcomes					
28‐ day all ‐ cause mortality	1257 (32.3)	288 (22.6)	400 (31.5)	569 (42.3)	< 0.001

Abbreviations: ACAG, albumin corrected anion gap; AECOPD, acute exacerbation of chronic obstructive pulmonary disease; ARF, acute respiratory failure; APSIII, acute physiology score III; BMI, body mass index; CHF, congestive heart failure; DM, diabetes mellitus; HA, human albumin; HB, hemoglobin; HTN, hypertension; IMV, invasive mechanical ventilation; OASIS, oxford acute severity of illness score; PaCO_2_, partial pressure of carbon dioxide in arterial blood; PaO_2_, partial pressure of oxygen in arterial blood; PF ratio, PaO_2_/FiO_2_ ratio; pH, potential of hydrogen; Plt, platelet; SAPSII, simplified acute physiology score II; SOFA, sequential organ failure assessment; T, tertiles; WBC, white blood cell count.

### 3.2 Outcome

The overall 28‐day all‐cause mortality was 32.3%. The 28‐day all‐cause mortality rates were 22.6%, 31.5%, and 45.3% for patients in the low, intermediate, and high ACAG tertiles, respectively.

### 3.3 ACAG and 28‐Day all‐Cause Mortality

Multivariable Cox regression analyses were used to evaluate the association between ACAG and 28‐day all‐cause mortality (Table 2). In an unadjusted model, ACAG demonstrated a positive association with 28‐day all‐cause mortality (HR 1.057, 95% CI 1.050–1.065, *p* < 0.001). In a model minimally adjusted for age, gender, BMI, and race, ACAG demonstrated a positive association with 28‐day all‐cause mortality (HR 1.062, 95% CI 1.054–1.070, *p* < 0.001). This relationship remained consistent when ACAG was presented as a continuous variable (HR 1.037, 95% CI 1.025–1.048, *p* < 0.001) after adjusting for all potential confounders (Model IV) (Table 2). Upon inclusion of ACAG as a categorized variable in the fully adjusted model, the changing trend of the effect size across different ACAG groups exhibited non‐equidistance. The risk of 28‐day all‐cause mortality was 1.244 times higher in the middle ACAG tertiles compared with the low tertiles (HR 1.244, 95% CI 1.062 ~ 1.457, *p* < 0.001), and a 1.483‐fold higher risk of mortality was observed in the high ACAG tertiles compared with the low tertiles (HR 1.483, 95% CI 1.244 ~ 1.768, *p* < 0.001), with a significant trend (*p* < 0.001) (Table 2). We have considered several other models that account for ‘HA infusion during the first 2 days of ICU admission,’ ‘HA infusion during the first 3 days of ICU admission,’ and ‘HA infusion throughout the ICU stay.’ The results from these additional models are also consistent with our primary analysis, as shown in Supplementary Table 1.

**TABLE 2:** Associations between ACAG and 28‐day all‐cause mortality in the multivariable Cox regression model.

Model	ACAG (*n* = 3888)		T1 (*n* = 1274)	T2 (*n* = 1270)	T3 (*n* = 1344)	*P* for trend
	HR (95%CI)	*p*	HR (95%CI)	HR (95%CI)	HR (95%CI)	
Crude model	1.057 (1.050–1.065)	< 0.001	1.000 (Ref)	1.479 (1.272–1.721)	2.234 (1.939–2.574)	< 0.001
Model I	1.062 (1.054–1.070)	< 0.001	1.000 (Ref)	1.406 (1.208–1.636)	2.226 (1.932–2.566)	< 0.001
Model II	1.025 (1.016–1.035)	< 0.001	1.000 (Ref)	1.143 (0.979–1.334)	1.281 (1.093–1.501)	0.002
Model III	1.031 (1.019–1.043)	< 0.001	1.000 (Ref)	1.182 (1.009–1.384)	1.348 (1.130–1.609)	< 0.001
Model IV	1.037 (1.025–1.048)	< 0.001	1.000 (Ref)	1.244 (1.062–1.457)	1.483 (1.244–1.768)	< 0.001

*Note:* Crude model, no other covariates were adjusted.

Abbreviations: ACAG, albumin corrected anion gap; AECOPD, acute exacerbation of chronic obstructive pulmonary disease; APSIII, acute physiology score III; CHF, congestive heart failure; CI, confidence interval; DM, diabetes mellitus; HA, human albumin; HB, hemoglobin; HR, hazard ratio; HTN, hypertension; ICU, intensive care unit; IMV, invasive mechanical ventilation; OASIS, Oxford acute severity of illness score; PaCO2, partial pressure of carbon dioxide in arterial blood; PaO2, partial pressure of oxygen in arterial blood; PF ratio, PaO2/FiO2 ratio; pH, potential of hydrogen; Plt, platelet; Ref, reference; SAPSII, simplified acute physiology score II; SOFA, sequential organ failure assessment; T, tertiles; WBC, white blood cell.

Model I, we adjusted age, gender, BMI, and race.

Model II, we adjusted Model I plus CHF, AECOPD, DM, HTN, APSIII, SAPSII, OASIS, and SOFA.

Model III, we adjusted Model II plus HB, WBC, Plt, glucose, creatinine, lactate, pH, PaO_2_, PaCO_2_, PF ratio, IMV, and vasoactive agent.

Model IV, we adjusted Model III plus infusion HA on the first day of ICU admission.

### 3.4 The Linear Association Between ACAG and 28‐Day all‐Cause Mortality

**FIGURE 2:**
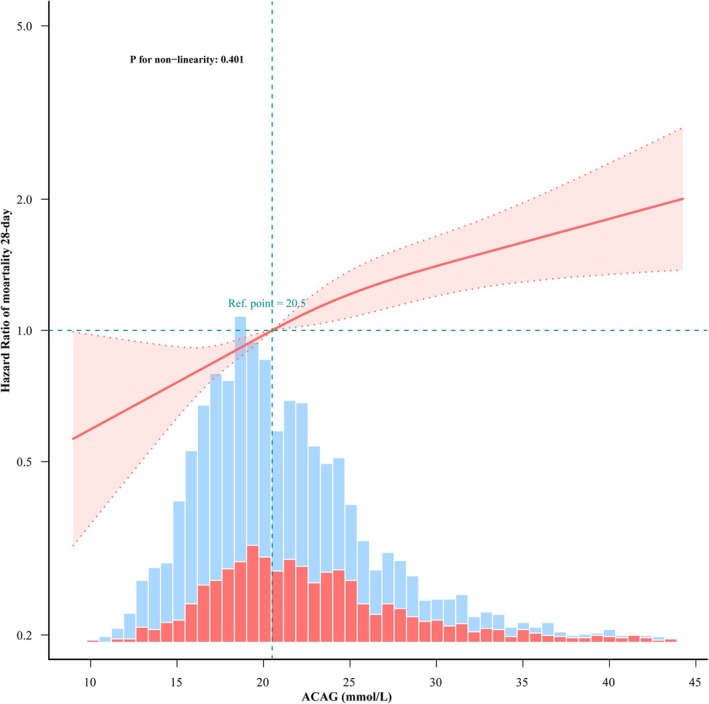
Smooth curve fitting: association between ACAG and all‐cause mortality of 28‐day. *Note:* This figure demonstrates multivariable adjusted HRs for all‐cause mortality of 28‐day in ICU according to levels of ACAG on a continuous scale (mmol/L). Solid deep red lines are multivariable‐adjusted HRs. Light red areas are the 95% CI derived from restricted cubic spline regressions with four knots. Dashed black lines are reference lines for no association at a hazard ratio of 1.0. All‐cause mortality of 28‐day is increased as ACAG concentration increased. Only 99% of the data is shown. All‐cause mortality of 28‐day analysis is adjusted age, gender, race, BMI, CHF, AECOPD, DM, HTN, APSIII, SAPII, OASIS, SOFA, HB, WBC, Plt, glucose, creatinine, lactate, pH, PaO_2_, PaCO_2_, PF ratio, IMV, vasoactive agent, and HA infusion.

A linear dose–response relationship between ACAG and 28‐day all‐cause mortality was noted following adjustment for gender, age, race, BMI, CHF, AECOPD, DM, HTN, APSIII, SAPSII, OASIS, SOFA, HB, WBC, Plt, glucose, creatinine, lactate, pH, PaO_2_, PaCO_2_, PF ratio, IMV, vasoactive agent, and infusion HA, as shown in Figure 2.

### 3.5 Kaplan–Meier Survival Curve Analysis

**FIGURE 3:**
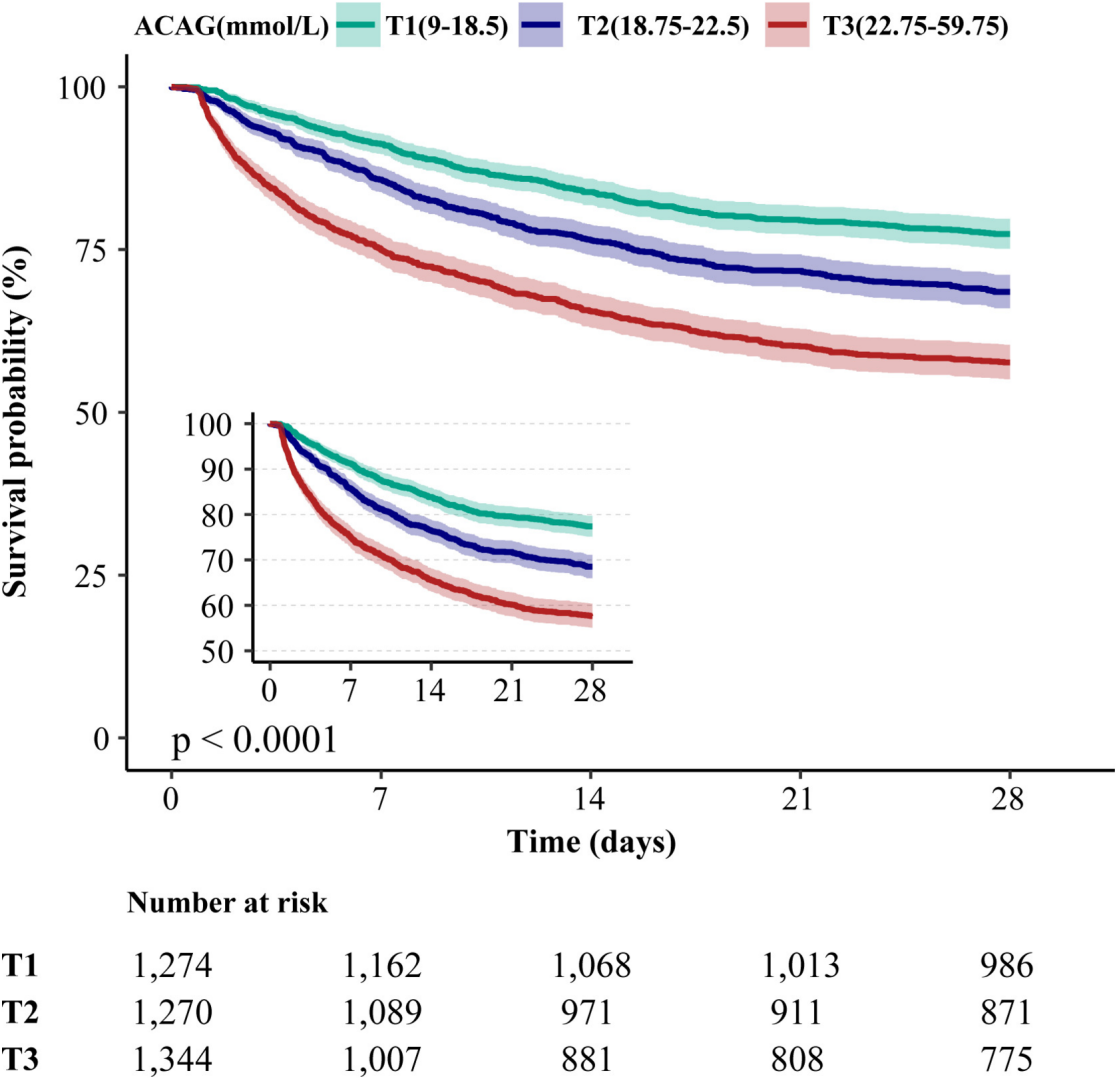
*Note:* Kaplan–Meier survival curve of mortality of 28‐day in ICU among tertiles groups of ACAG. ACAG, albumin corrected anion gap; ICU, intensive care unit; T, tertiles.

The Kaplan–Meier curve is illustrated in Figure 3. The T1 group has a higher survival rate than the T2 and T3 groups, respectively. The survival rate of the T3 group is the lowest. The log‐rank test shows a significant result, *p* < 0.0001.

### 3.6 Sensitivity Analysis

In the sensitivity analysis, we excluded 2,178 ARF patients with incomplete covariate variables data. Considering the relationship between ACAG and all‐cause mortality of 28‐day, similar findings were observed (Supplementary Table 2).

### 3.7 Subgroup Analysis

**FIGURE 4:**
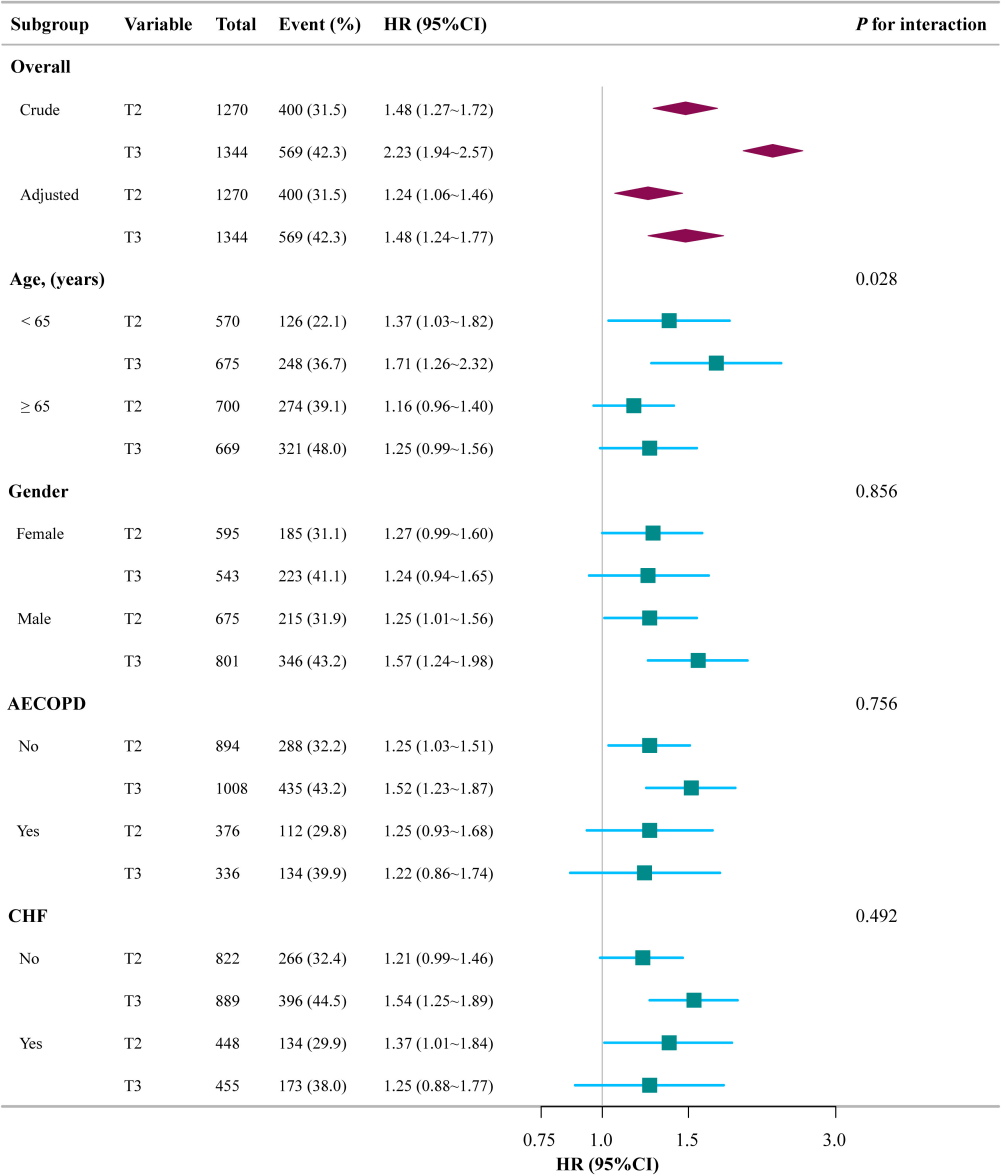
Subgroup analyses of the association between ACAG and 28‐day all‐cause mortality with different characteristics. Except for the stratification component itself, each stratification factor was adjusted for gender, age, race, BMI, AECOPD, CHF, DM, HTN, APSIII, SAPSII, OASIS, SOFA, HB, WBC, Plt, glucose, creatinine, lactate, pH, PaO_2_, PaCO_2_, PF ratio, IMV, vasoactive agent, and infusion HA. ACAG, albumin corrected anion gap; AECOPD, acute exacerbation of chronic obstructive pulmonary disease; APSIII, acute physiology score III; CHF, congestive heart failure; CI, confidence interval; DM, diabetes mellitus; HA, human albumin; HB, hemoglobin; HR, hazard ratio; HTN hypertension; IMV, invasive mechanical ventilation; OASIS, oxford acute severity of illness score; PaCO_2_, partial pressure of carbon dioxide in arterial blood; PaO_2_, partial pressure of oxygen in arterial blood; PF ratio, PaO_2_/FiO_2_ ratio; pH, potential of hydrogen; Plt, platelet; Ref, reference; SAPSII, simplified acute physiology score II; SOFA, sequential organ failure assessment; T, tertiles; WBC, white blood cell.

Subgroup analyses were conducted to assess the relationship between elevated ACAG levels and 28‐day all‐cause mortality, as depicted in Figure 4. After stratifying by several subgroups (age, gender, AECOPD [Yes/No], CHF [Yes/No]), no significant interactions were observed in any of the subgroups. Although a significant interaction was noted in the subgroup analysis between age < 65 years and age ≥ 65 years (*p* = 0.023), the effect size of ACAG on 28‐day all‐cause mortality remained similar across both subgroups.

## 4 Discussion

ARF carries a significant risk of occurrence and is linked to a high mortality rate [16]. The annual mortality rate has witnessed a rise of 3.4%. It continues to stand as one of the primary reasons for hospitalization [17]. The AG is a parameter that can be determined mathematically, based on the concentration difference between cations and anions, and reflects the unmeasured anion. It serves as a parameter for assessing acid–base status and aids in determining different forms of metabolic acidosis.

Compared with AG, after its correction for the effect of albumin, ACAG can more accurately determine occult organizations [18], be more conducive to the diagnosis of high lactic acidosis [19], and more accurately determine the type of acid–base metabolism turbulence. ARF, particularly during severe hypoxia, can lead to cellular hypoxia and the production of various metabolic byproducts, triggering metabolic acidosis and increasing AG. The presence of hypoalbuminemia in ICU patients with ARF frequently results in underestimated AG. Nevertheless, the relationship between ACAG and 28‐day all‐cause mortality in critically ill patients with ARF in the ICU is understudied. Therefore, our research seeks to explore the association between ACAG and 28‐day all‐cause mortality in ICU patients diagnosed with ARF.

In this retrospective cohort study, 3888 patients with ARF were included, revealing an independent association between ACAG and 28‐day all‐cause mortality. In the current study, we observed that a rising ACAG was linked to a significantly elevated risk of 28‐day all‐cause mortality in ICU patients diagnosed with ARF. The identified association persisted independently of several vital covariates and confounding factors. Furthermore, the variation trend of the effective value in different ACAG groups was non‐equidistant. Through a generalized additive model, we observed a linear association between ACAG and the 28‐day all‐cause mortality of patients diagnosed with ARF. A recent study reported that in seriously AKI patients receiving CRRT in the ICU, an elevated ACAG level (> 20 mmol/L) at the start of treatment was associated with all‐cause mortality; this indicates the ACAG can serve as a potential indicator for these patients' adverse consequences [20]; consistent with our study's findings, a linear relationship was observed between the ACAG at Continuous Renal Replacement Therapy (CRRT) initiation and all‐cause mortality in the ICU. In contrast, patients suffering cardiac arrest who had excessive ACAG (> 20 mmol/L) had a greater in‐hospital all‐cause mortality [21]; the probability of all‐cause death in hospitalized patients experiencing cardiac arrest showed a positive correlation with the ACAG upon ICU admission, albeit in a nonlinear fashion. High serum ACAG levels were a significant risk factor for 30‐day all‐cause mortality in critically ill patients with acute myocardial infarction; ACAG concentration and 30‐day all‐cause mortality had a nonlinear relationship [22]. Albumin constitutes a substantial portion of the unmeasured anions, and alterations in albumin levels can induce fluctuations in the AG level. The serum albumin concentration typically experiences a marked decline in the early stages of a critical illness, and any subsequent increase is typically observed during the recovery phase of the condition [23].

We acknowledge that ARF is a highly heterogeneous condition with various etiologies, including but not limited to AECOPD and CHF. The pathophysiological mechanisms, clinical manifestations, and responses to treatment can vary significantly depending on the underlying cause. For example, ARF caused by AECOPD is primarily associated with airway inflammation and airflow limitation [17]. In contrast, ARF caused by CHF is more related to pulmonary circulation disorders and pulmonary edema [11]. In our study, we conducted subgroup analyses to explore the relationship between ACAG and 28‐day all‐cause mortality across different etiologies of ARF. The results showed that the positive correlation between ACAG and mortality remained stable across all subgroups, indicating that ACAG, as a comprehensive indicator, can effectively predict short‐term mortality risk in ARF patients with diverse etiologies. This finding is consistent with the clinical subphenotyping approach mentioned in the literature [24], further supporting the robustness of our observed associations in different subgroups.

The clustering structure identification method based on ordering points (OPTICS) and the consensus *K* clustering method mentioned in reference [24] provide important references for the subphenotype research of ARF. These methods can effectively identify the natural clustering structures within the data and enhance the stability of clustering results through multiple iterations and consistency assessments. Future studies may consider employing OPTICS and consensus *K* clustering, in conjunction with larger sample sizes and more detailed clinical data, to further explore the heterogeneity of ARF.

The current study has several limitations that should be considered. Our conclusions are limited to ARF patients with an ICU stay exceeding 24 hours, and the study design is observational, which may be influenced by uncontrolled confounding variables. We assessed ACAG statically over a 30‐hours interval, which limits our ability to fully capture its dynamic nature and its potential impact on patient outcomes over time. Future research should focus on longitudinal measurements of ACAG to better understand its impact on outcomes. The MIMIC database is a single‐center dataset from the BIDMC, which restricts the external validity of our results. Our findings may not apply directly to other populations or healthcare systems with different demographics, practices, or policies. Future prospective, multicenter studies are needed to confirm the generalizability and to explore the causal relationship between ACAG and 28‐day mortality in ARF patients in the ICU.

## 5 Conclusions

Among ICU patients with ARF, an elevated ACAG was linked to an increased risk of 28‐day all‐cause mortality. A linear positive correlation manifested between ACAG and the 28‐day mortality rate for all causes.

## Author Contribution

Study concept and design: JQ, YZ. Acquisition, analysis, or interpretation of data: JQ, XT. Drafting of the manuscript: JQ, WX. Critical revision of the manuscript for important intellectual content: all authors. Statistical analysis: JQ, XT. Administrative, technical, or material support: all authors. Study supervision: JQ, WX, YZ. Final approval of the article: all authors. Guarantor: JQ, YZ.

## Ethics Statement

The research involving human participants was reviewed and approved by the Institutional Review Board of the Massachusetts Institute of Technology and the Beth Israel Deaconess Medical Center. In accordance with national legislation and institutional requirements, written informed consent from the participants was not required for this study.

## Consent

Not applicable.

## Conflicts of Interest

All authors declare no conflicts of interest.

## Supporting information


**Table S1.** Associations between ACAG and 28‐day all‐cause mortality in the multivariable Cox regression model: analysis including various albumin infusion timings during ICU stay.

**Table S2.** Associations between ACAG and 28‐day all‐cause mortality in the multivariable Cox regression model: analysis excluding data with missing covariates.

## Data Availability

The data that support the findings of this study are available from the corresponding author upon reasonable request.

## References

[1] S. Fujishima , “Guideline‐Based Management of Acute Respiratory Failure and Acute Respiratory Distress Syndrome,” Journal of intensive care 11, no. 1 (2023): 10, 10.1186/s40560-023-00658-3. 36895001 PMC9998250

[2] M. Park , S. J. Jung , S. Yoon , J. M. Yun , and H. J. Yoon , “Association Between the Markers of Metabolic Acid Load and Higher all‐Cause and Cardiovascular Mortality in a General Population With Preserved Renal Function,” Hypertension Research 38, no. 6 (2015): 433–438, 10.1038/hr.2015.23. 25762414

[3] A. A. Nanji , “Decreased Anion Gap Associated With Hypoalbuminemia and Polyclonal Gammopathy,” JAMA 246, no. 8 (1981): 859, 10.1001/jama.1981.03320080045027. 6166764

[4] A. Sheinenzon , M. Shehadeh , R. Michelis , E. Shaoul , and O. Ronen , “Serum Albumin Levels and Inflammation,” International journal of biological macromolecules 184 (2021): 857–862, 10.1016/j.ijbiomac.2021.06.140. 34181998

[5] B. Pratumvinit , L. Lam , N. Kongruttanachok , et al., “Anion Gap Reference Intervals Show Instrument Dependence and Weak Correlation With Albumin Levels,” Clinica Chimica Acta 500 (2020): 172–179, 10.1016/j.cca.2019.10.012. 31669932

[6] Q. Zhou , Y. Miao , P. Wang , F. Li , J. Li , and N. Li , “Association Between Albumin Corrected Anion Gap and Mortality in Septic Older Adults,” Geriatric Nursing 60 (2024): 580–585, 10.1016/j.gerinurse.2024.10.022. 39461109

[7] K. Berend , A. P. J. de Vries , and R. O. B. Gans , “Physiological Approach to Assessment of Acid‐Base Disturbances,” New England Journal of Medicine 371, no. 15 (2014): 1434–1445, 10.1056/NEJMra1003327. 25295502

[8] P. Li , L. Shi , X. Yan , et al., “Albumin Corrected Anion Gap and the Risk of In‐Hospital Mortality in Patients With Acute Pancreatitis: A Retrospective Cohort Study,” Journal of Inflammation Research 16 (2023): 2415–2422, 10.2147/JIR.S412860. 37313307 PMC10258038

[9] T. Hu , Z. Zhang , and Y. Jiang , “Albumin Corrected Anion Gap for Predicting in‐Hospital Mortality Among Intensive Care Patients With Sepsis: A Retrospective Propensity Score Matching Analysis,” Clinica Chimica Acta 521 (2021): 272–277, 10.1016/j.cca.2021.07.021. 34303712

[10] A. E. W. Johnson , L. Bulgarelli , L. Shen , et al., “MIMIC‐IV, a Freely Accessible Electronic Health Record Dataset,” Scientific data 10, no. 1 (2023): 1–10, 10.1038/s41597-022-01899-x. 36596836 PMC9810617

[11] Q. Yang , J. Zheng , X. Chen , et al., “Relationship Between Driving Pressure and Mortality in Ventilated Patients With Heart Failure: A Cohort Study,” Canadian Respiratory Journal 2021 (2021): 1–8, 10.1155/2021/5574963. PMC8648448 34880958

[12] B. J. Jones and P. J. Twomey , “The Anion Gap Revisited,” International journal of clinical practice 63, no. 10 (2009): 1409–1412, 10.1111/j.1742-1241.2009.02074.x. 19769697

[13] B. Zhao , Y. Li , X. Lang , et al., “Increased Serum Albumin Corrected Anion gap Levels Are Associated With Increased Incidence of New‐Onset HF and Poor Prognosis in Patients With Acute Myocardial Infarction,” Clinica Chimica Acta 544 (2023): 117354, 10.1016/j.cca.2023.117354. 37076098

[14] R. J. A. Little and D. B. Rubin , Statistical Analysis With Missing Data, 1st ed. (Wiley, 2002), 10.1002/9781119013563.

[15] R. Wang , J. Li , H. Chen , et al., “Preoperative Albumin Corrected Anion gap Is Associated With In‐Hospital and Long‐Term Mortality in Patients Undergoing Coronary Artery Bypass Grafting in a Retrospective Cohort Study,” Journal of thoracic disease 14, no. 12 (2022): 4894–4903, 10.21037/jtd-22-1633. 36647463 PMC9840039

[16] Q. Yang , J. Zheng , W. Chen , et al., “Association Between Preadmission Metformin Use and Outcomes in Intensive Care Unit Patients With Sepsis and Type 2 Diabetes: A Cohort Study,” Frontiers in medicine 8 (2021): 640785, 10.3389/fmed.2021.640785. 33855034 PMC8039324

[17] B. N. Burton , S. Trivedi , A. Beletsky , et al., “The Influence of Hospital Urbanicity on Mortality in Patients With Acute Respiratory Failure: A National Cohort Retrospective Analysis,” Respiratory Care: a monthly science journal 66, no. 12 (2021): 1789–1796, 10.4187/respcare.09003. PMC10408415 34548408

[18] V. Parcha , R. Kalra , S. P. Bhatt , L. Berra , G. Arora , and P. Arora , “Trends and Geographic Variation in Acute Respiratory Failure and ARDS Mortality in the United States,” Chest 159, no. 4 (2021): 1460–1472, 10.1016/j.chest.2020.10.042. 33393472 PMC7581392

[19] M. Hatherill , “Correction of the Anion Gap for Albumin in Order to Detect Occult Tissue Anions in Shock,” Archives of Disease in Childhood 87, no. 6 (2002): 526–529, 10.1136/adc.87.6.526. 12456555 PMC1755806

[20] L. S. Chawla , S. Shih , D. Davison , C. Junker , and M. G. Seneff , “Anion Gap, Anion Gap Corrected for Albumin, Base Deficit and Unmeasured Anions in Critically Ill Patients: Implications on the Assessment of Metabolic Acidosis and the Diagnosis of Hyperlactatemia,” BMC Emergency Medicine 8 (2008): 18, 10.1186/1471-227X-8-18. 19087326 PMC2644323

[21] L. Zhong , B. Xie , X. W. Ji , and X. H. Yang , “The Association Between Albumin Corrected Anion gap and ICU Mortality in Acute Kidney Injury Patients Requiring Continuous Renal Replacement Therapy,” Internal and Emergency Medicine 17, no. 8 (2022): 2315–2322, 10.1007/s11739-022-03093-8. 36112320 PMC9652260

[22] L. Jian , Z. Zhang , Q. Zhou , X. Duan , H. Xu , and L. Ge , “Association Between Albumin Corrected Anion Gap and 30‐Day all‐Cause Mortality of Critically ill Patients With Acute Myocardial Infarction: A Retrospective Analysis Based on the MIMIC‐IV Database,” BMC Cardiovascular Disorders 23, no. 1 (2023): 211, 10.1186/s12872-023-03200-3. 37118662 PMC10148465

[23] J. P. Nicholson , M. R. Wolmarans , and G. R. Park , “The Role of Albumin in Critical Illness,” British journal of anaesthesia 85, no. 4 (2000): 599–610, 10.1093/bja/85.4.599. 11064620

[24] J. Yang , B. Zhang , C. Hu , et al., “Identification of Clinical Subphenotypes of Sepsis After Laparoscopic Surgery,” Laparoscopic, Endoscopic and Robotic Surgery 7, no. 1 (2024): 16–26, 10.1016/j.lers.2024.02.001.

