# Supplementary material for: Association Between Albumin Corrected Anion Gap and 28‐Day All‐Cause Mortality in Patients With Acute Respiratory Failure in ICU: A Retrospective Study Based on the MIMIC‐IV Database
Source: Clin Respir J. 2025 Jul 9;19(7):e70100. doi: 10.1111/crj.70100 (PMC12238893; doi:10.1111/crj.70100)
Supplement: Supplementary file 1 — Table S1. Associations between ACAG and 28‐day all‐cause mortality in the multivariable Cox regression model: analysis including various albumin infusion timings during ICU stay. [file CRJ-19-e70100-s002.docx]

**Supplementary Table 1** Associations between ACAG and 28-day all-cause mortality in the multivariable Cox regression model: analysis including various albumin infusion timings during ICU stay

| Model | ACAG (n=3,888) | | T1 (n=1,274) | T2 (n=1,270) | T3 (n=1,344) | *P* for trend |
| --- | --- | --- | --- | --- | --- | --- |
|  | HR (95%CI) | *P* value | HR (95%CI) | HR (95%CI) | HR (95%CI) |  |
| Crude Model | 1.057 (1.050~1.065) | <0.001 | 1.00 (Ref) | 1.479 (1.272~1.721) | 2.234 (1.939~2.574) | <0.001 |
| Model I | 1.062 (1.054~1.070) | <0.001 | 1.00 (Ref) | 1.406 (1.208~1.636) | 2.226 (1.932~2.566) | <0.001 |
| Model II | 1.025 (1.016~1.035) | <0.001 | 1.00 (Ref) | 1.126 (0.965~1.315) | 1.264 (1.078~1.482) | 0.003 |
| Model III | 1.031 (1.019~1.043) | <0.001 | 1.00(Ref) | 1.182 (1.009~1.384) | 1.348 (1.130~1.609) | <0.001 |
| Model IV | 1.037 (1.025~1.048) | <0.001 | 1.00 (Ref) | 1.244 (1.062~1.457) | 1.483 (1.244~1.768) | <0.001 |
| Model IVa | 1.034 (1.022~1.046) | <0.001 | 1.00 (Ref) | 1.198 (1.022~1.404) | 1.403 (1.175~1.676) | <0.001 |
| Model IVb | 1.034 (1.021~1.045) | <0.001 | 1.00 (Ref) | 1.199 (1.023~1.405) | 1.403 (1.175~1.675) | <0.001 |
| Model IVc | 1.031 (1.019~1.043) | <0.001 | 1.00 (Ref) | 1.190 (1.015~1.394) | 1.377 (1.151~1.648) | <0.001 |

**Notes:**

Crude Model, no other covariates were adjusted.

Model I, we adjusted age, gender, BMI, and race.

Model II, we adjusted Model I plus CHF, AECOPD, DM, HTN, APSIII, SAPIII, OASIS, and SOFA.

Model III, we adjusted Model II plus HB, WBC, Plt, glucose, creatinine, lactate, pH, PaO_2_, PaCO_2_, PF ratio, IMV, vasoactive agent.

Model IV, we adjusted Model III plus HB, WBC, Plt, glucose, creatinine, lactate, pH, PaO_2_, PaCO_2_, PF ratio, IMV, vasoactive agent and HA infusion on the first day of ICU admission.

Model IVa, we adjusted Model III plus HB, WBC, Plt, glucose, creatinine, lactate, pH, PaO_2_, PaCO_2_, PF ratio, IMV, vasoactive agent, and HA infusion during the first two days of ICU admission.

Model IVb, we adjusted Model III plus HB, WBC, Plt, glucose, creatinine, lactate, pH, PaO_2_, PaCO_2_, PF ratio, IMV, vasoactive agent, and HA infusion during the first three days of ICU admission.

Model IVc, we adjusted Model III plus HB, WBC, Plt, glucose, creatinine, lactate, pH, PaO_2_, PaCO_2_, PF ratio, IMV, vasoactive agent, and HA infusion during the ICU stay.

**Abbreviations:**

ACAG, albumin corrected anion gap; HR, hazard ratio; CI, confidence interval; T, tertiles; Ref, reference; CHF, congestive heart failure; AECOPD, acute exacerbation of chronic obstructive pulmonary disease; DM, diabetes mellitus; HTN hypertension; APSIII, acute physiology score III; SAPSII, simplified acute physiology score II; OASIS, oxford acute severity of illness score; SOFA, sequential organ failure assessment; HB, hemoglobin; WBC, white blood cell; Plt, platelets; pH, potential of hydrogen; PaO_2_, partial pressure of oxygen in arterial blood; PaCO_2_, partial pressure of carbon dioxide in arterial blood; PF ratio, PaO_2_/FiO_2_ ratio; IMV, invasive mechanical ventilation; HA, human albumin; ICU, intensive care unit.
