# Supplementary material for: Association Between Albumin Corrected Anion Gap and 28‐Day All‐Cause Mortality in Patients With Acute Respiratory Failure in ICU: A Retrospective Study Based on the MIMIC‐IV Database
Source: Clin Respir J. 2025 Jul 9;19(7):e70100. doi: 10.1111/crj.70100 (PMC12238893; doi:10.1111/crj.70100)
Supplement: Supplementary file 2 — Table S2. Associations between ACAG and 28‐day all‐cause mortality in the multivariable Cox regression model: analysis excluding data with missing covariates. [file CRJ-19-e70100-s001.docx]

| Model | ACAG (n=1,710) | | T1 (n=441) | T2 (n=561) | T3 (n=708) | *P* for trend |
| --- | --- | --- | --- | --- | --- | --- |
|  | HR (95% CI) | *P* value | HR (95% CI) | HR (95% CI) | HR (95% CI) |  |
| Crude Model | 1.051 (1.040~1.061) | <0.001 | 1.00 (Ref) | 1.445 (1.132~1.884) | 2.204 (1.759~2.762) | <0.001 |
| Model I | 1.055 (1.044~1.066) | <0.001 | 1.00 (Ref) | 1.437 (1.126~1.833) | 2.161 (1.724~2.708) | <0.001 |
| Model II | 1.034 (1.021~1.048) | <0.001 | 1.00 (Ref) | 1.233 (0.962~1.579) | 1.457 (1.134~1.873) | 0.003 |
| Model III | 1.031 (1.015~1.048) | <0.001 | 1.00 (Ref) | 1.205 (0.936~1.551) | 1.327 (1.007~1.749) | 0.049 |
| Model IV | 1.031 (1.015~1.048) | <0.001 | 1.00 (Ref) | 1.250 (0.984~1.587) | 1.265 (0.973~1.644) | 0.079 |

**Supplementary Table 2** Associations between ACAG and 28-day all-cause mortality in the multivariable Cox regression model: analysis excluding data with missing covariates.

**Notes**:

Crude Model, no other covariates were adjusted.

Model I, we adjusted age, gender, BMI, and race.

Model II, we adjusted Model I plus CHF, AECOPD, DM, HTN, APSIII, SAPSII, OASIS, and SOFA.

Model III, we adjusted Model II plus HB, WBC, Plt, glucose, creatinine, lactate, pH, PaO_2_, PaCO_2_, PF ratio, IMV, vasoactive agent.

Model IV, we adjusted Model III plus infusion HA on the first day of ICU admission.

**Abbreviations:**

ACAG, Albumin corrected anion gap; HR, hazard ratio; CI, confidence interval; T, tertiles; Ref, reference; CHF, congestive heart failure; AECOPD, acute exacerbation of chronic obstructive pulmonary disease; DM, diabetes mellitus; HTN hypertension; APSIII, acute physiology score III; SAPSII, simplified acute physiology score II; OASIS, oxford acute severity of illness score; SOFA, sequential organ failure assessment; HB, hemoglobin; WBC, white blood cell; Plt, platelets; pH, potential of hydrogen; PaO_2_, partial pressure of oxygen in arterial blood; PaCO_2_, partial pressure of carbon dioxide in arterial blood; PF ratio, PaO_2_/FiO_2_ ratio; IMV, invasive mechanical ventilation; HA, human albumin; ICU, intensive care unit.
